# Evaluation of a commercial multi-dimensional echocardiography technique for ventricular volumetry in small animals

**DOI:** 10.1186/s12947-018-0128-9

**Published:** 2018-07-03

**Authors:** Jana Grune, Annelie Blumrich, Sarah Brix, Sarah Jeuthe, Cathleen Drescher, Tilman Grune, Anna Foryst-Ludwig, Daniel Messroghli, Wolfgang M. Kuebler, Christiane Ott, Ulrich Kintscher

**Affiliations:** 1 Institute of Pharmacology, Center for Cardiovascular Research, Charité -Universitaetsmedizin Berlin, Hessische Str. 3-4, 10115 Berlin, Germany; 2 German Center for Cardiovascular Research (DZHK), partner site Berlin, 10117 Berlin, Germany; 3 Department of Molecular Toxicology, German Institute of Human Nutrition Potsdam-Rehbruecke (DIfE), 14558 Nuthetal, Germany; 4 German Center for Diabetes Research (DZD), 85764 Muenchen-Neuherberg, Germany; 5 Internal Medicine/Cardiology, Deutsches Herzzentrum Berlin, Augustenburger Platz 1, 13353 Berlin, Germany; 6 Department of Cardiology, Charité University Medicine Berlin, Augustenburger Platz 1, 13353 Berlin, Germany; 7 Institute of Physiology, Charité University Medicine Berlin, Charitéplatz 1, 10117 Berlin, Germany

**Keywords:** 3D echocardiography, Heart failure, Volumetry, Preclinical imaging, Small animals

## Abstract

**Background:**

The assessment of ventricular volumes using conventional echocardiography methods is limited with regards to the need of geometrical assumptions. In the present study, we aimed to evaluate a novel commercial system for three-dimensional echocardiography (3DE) in preclinical models by direct comparison with conventional 1D- and 2D-echocardiography (1DE; 2DE) and the gold-standard technique magnetic resonance imaging (MRI). Further, we provide a standard operating protocol for image acquisition and analysis with 3DE.

**Methods:**

3DE was carried out using a 30 MHz center frequency transducer coupled to a Vevo®3100 Imaging System. We evaluated under different experimental conditions: 1) in vitro phantom measurements served as controlled setting in which boundaries were clearly delineated; 2) a validation cohort composed of healthy C57BL/6 J mice and New Zealand Obese (NZO) mice was used in order to validate 3DE against cardiac MRI; 3) a standard mouse model of pressure overload induced-heart failure was investigated to estimate the value of 3DE.

**Results:**

First, in vitro volumetry revealed good agreement between 3DE assessed volumes and the MRI-assessed volumes. Second, cardiac volume determination with 3DE showed smaller mean differences compared to cardiac MRI than conventional 1DE and 2DE. Third, 3DE was suitable to detect reduced ejection fractions in heart failure mice. Fourth, inter- and intra-observer variability of 3DE showed good to excellent agreement regarding absolute volumes in healthy mice, whereas agreement rates for the relative metrics ejection fraction and stroke volume demonstrated good to moderate observer variabilities.

**Conclusions:**

3DE provides a novel method for accurate volumetry in small animals without the need for spatial assumptions, demonstrating a technique for an improved analysis of ventricular function. Further validation work and highly standardized image analyses are required to increase reproducibility of this approach.

**Electronic supplementary material:**

The online version of this article (10.1186/s12947-018-0128-9) contains supplementary material, which is available to authorized users.

## Background

Echocardiography provides a reliable, cost-effective and widely available technique for evaluation of cardiac function in both human and small animal imaging. However, the assessment of cardiac wall- and chamber dimensions by conventional echocardiography methods is limited with regards to the need of geometrical assumptions for formula-based computation of three-dimensional volumes [1, 2]. Therefore, cardiac magnetic resonance imaging (MRI) is considered as gold standard measurement for left ventricular (LV) volumetry even in rodents, since it allows the assessment of the entire heart in multiple planes [3, 4]. Nevertheless, high expenses and consecutively restricted availability of MRI together with a time-consuming image acquisition process limit its widespread application [3–6]. Recently, matrix array transducers for clinical three-dimensional echocardiography (3DE) have been developed that allow “real-time” volumetry with a superior precision compared to two-dimensional echocardiography (2DE) [7]. Albeit limitations are comparable between clinical and small animal echocardiography, a comprehensive evaluation of a novel, commercially available 3DE in small animals is currently lacking.

Due to their high temporal resolution, linear M-Mode measurements (one-dimensional echocardiography; 1DE) have been widely applied in small animal imaging, especially to determine LV wall thicknesses and –mass [8, 9]. However, with respect to clinical guidelines, 1DE-derived volumetry is obsolete since it is highly vulnerable against misestimations, especially in case of asymmetric LV shape [2, 10]. Increased temporal and spatial resolutions of ultrasound transducers allow assessment of appropriate 2D B-Mode images of the LV and consecutive volumetry by method of disks [11, 12]. Several studies reported on tomographic reconstruction of such 2DE images as potential technique for 3DE in small animals [13, 14]. Albeit this experimental approach has been successfully validated against MRI [14, 15], its widespread application was limited with regards to procedural standardization, ECG-gated synchronization, a rather low spatial resolution and the lack of corresponding post-procession software.

**Fig. 1:**
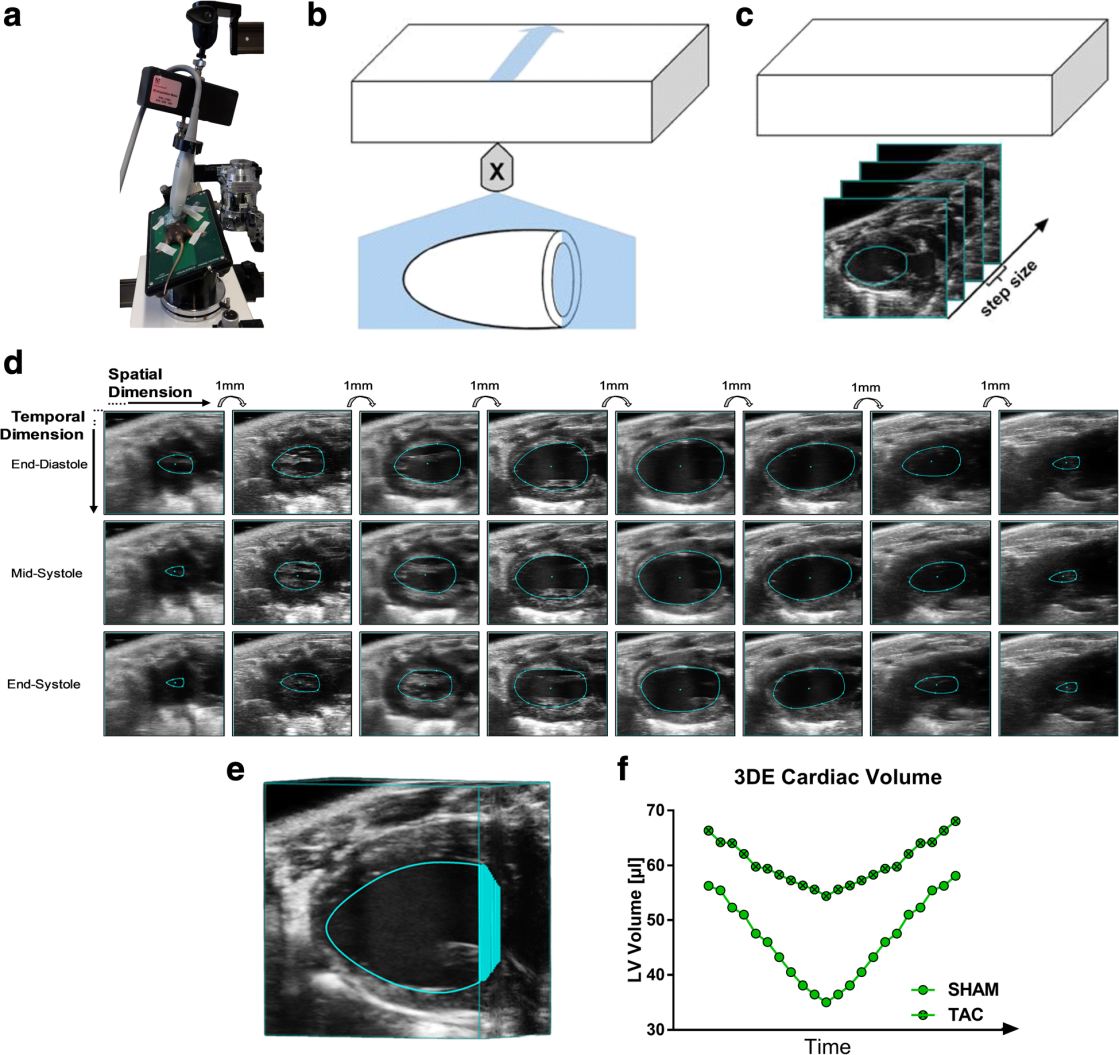
Concept of 3D-echocardiography in small animals. **a** 3D-motor installed on the transducer. **b** 3D-motor allows the transducer to move unidirectional realizing 3DE. **c** Recording different cardiac slices during the cardiac cycle. **d** Chronogram demonstrating the link between spatial (3D) and temporal dimension (4D). **e** Multi-slice reconstruction of 3DE. **f** 3D-volume tracking of exemplary SHAM and TAC-mice along the cardiac cycle (4DE)

Recently, these pioneering studies paved the way for a commercially available rodent 3DE system that allows automated respiratory-gated acquisition of high-resolution 2D B-Mode images at different levels of the heart and at every point of the cardiac cycle (Fig. 1a-c) [16, 17]. 3DE data sets are built by tomographic multi-slice reconstruction of acquired 2D images up to a step size of 50 μm, and can be analyzed with a dedicated software package allowing visualization and calculation of LV volumes along the cardiac cycle (Fig. 1d-f).

In this study, we aimed to evaluate this automated commercial 3DE system against MRI and standard 2DE under different experimental conditions: 1) in vitro phantom measurements served as controlled setting in which boundaries were clearly delineated; 2) a validation cohort composed of healthy C57BL/6 J mice and New Zealand Obese (NZO) mice was used in order to validate 3DE against cardiac MRI as gold standard measurement; 3) a mouse model of pressure overload-induced heart failure was investigated to estimate the incremental value of 3DE for a standard application in the field of applied research.

## Materials and methods

### In vitro Volumetry

**Fig. 2:**
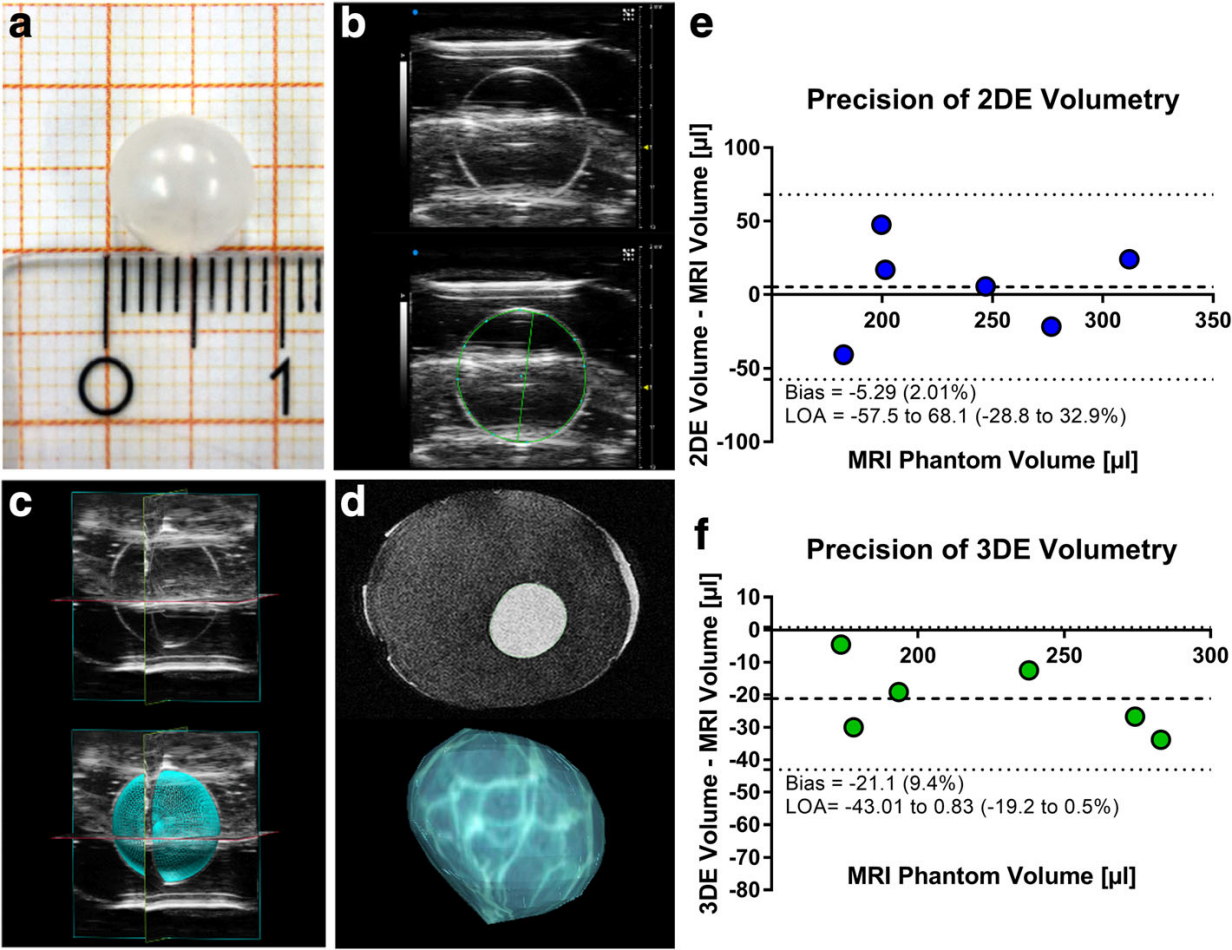
In vitro volumetry. **a** Photo of round-shaped phantom. Scale = 1 cm. **b** Exemplary 2DE, **c** 3DE, **d** and magnetic resonance images of phantoms with and without exemplary tracings and 3D reconstructions. **e** Bland-Altman analysis of 2DE and **f** 3DE volumes compared to gold standard magnetic resonance imaging (MRI) assessed phantom volumes*. n* = 6

Round-shaped, oval latex balloons between 0.6 and 1.0 cm in size (*n* = 6; 176–300 μL; Fig. 2a) mimicking mice hearts, served as phantoms for ultrasound- and MRI measurements, as described before [18]. Balloons were filled with tap water before being embedded in a 1% agarose gel matrix.

Phantoms were scanned in a 3 Tesla small animal magnetic resonance system (MR Solutions, Guildford, United Kingdom) with a quadrature birdcage cardiac volume coil as previously reported by us [19]. A T2-weighed fast spin echo sequence with following parameters was applied: repetition time, 4800 ms; echo time, 68 ms; flip angle, 90°; field of view, 40.00\40.00\ 0.30 mm; pixel spacing 0.16\0.16; number of signal averages, 3; slice thickness 0.3 mm. Volumes were calculated by multi-slice tracing using Osirix software (version 7.0.3; Pixmeo SARL, Geneva, Switzerland).

For 2DE, B-Mode images of the maximum dimension of the round-shaped phantoms were acquired and volumes were calculated using the monoplane method of disks.

3DE image acquisition was started at the maximum dimension of the phantom at a slice thickness of 0.3 mm (equivalent to MRI).

### Validation cohort

All animal procedures were performed in accordance with the guidelines of the German Law on the Protection of Animals and were approved by the local authorities (Landesamt für Gesundheit und Soziales, Berlin, Germany). Animals used in this study served as controls in ongoing projects and were kept under identical housing conditions (12 h light/dark cycle, standard diet ad libitum, 21 °C room temperature).

A cohort of 5 male C57BL/6 J and 5 male NZO mice (*n* = 10 total) was analyzed regarding cardiac volumes and ejection fraction (EF). All mice underwent echocardiography (1DE, 2DE and 3DE) and cardiac magnetic resonance (CMR) examination at the age of 22 weeks as described below. All data sets were acquired prospectively and analyzed for this study in a retrospective manner.

### Heart failure cohort

Male C57BL/6 J mice (8–9 weeks) were anesthetized by intra-peritoneal injection of ketamine/xylazine (100 mg/kg/d, 20 mg/kg/d) (Sigma-Aldrich, Steinheim, Germany) before partial sternotomy was performed. Transverse aortic constriction (TAC) was induced by placing a silk suture around the aorta between right and left carotid arteries and a 26 gauge needle as previously reported by us (*n* = 9) [20]. Same procedure was performed on SHAM-operated animals (*n* = 7) except for the aortic banding. Echocardiography (1DE, 2DE and 3DE) was performed 10 weeks after TAC or SHAM-surgery.

### CMR measurements in vivo

Similar to in vitro measurements, C57BL/6 J and NZO mice were scanned using a 3 Tesla small animal MRI system (MR Solutions, Guildford, United Kingdom) with a quadrature birdcage cardiac volume coil [19]. After induction of inhalative anesthesia with isoflurane-oxygen (4–5%) animals were positioned in a coil head first position and ECG electrodes were placed on the mice’ feet. Anesthesia was maintained throughout the examination via inhalation of 1–2% isoflurane-oxygen to achieve heart rates around 400 beats per minutes. Mice were positioned in a heat-controlled animal bed (Equipment Veterinaire Minerve, Esternay, France) to maintain body temperature at 37 °C. Images were acquired using respiratory and ECG-gated gradient-echo cine sequences resulting in a LV cine short-axis stack with five to eight short-axis planes completely covering the LV (phases, 16; repetition time, 10 ms; echo time, 3 ms; flip angle, 20°; field of view, 40.00\40.00\1.00 mm; pixel spacing 0.16\0.16 mm; number of signal averages, 3; slices, 8; slice thickness 1.0 mm). Cardiac volumes and ejection fraction (EF) were assessed using CMR42 software package (version 3.4.1; Circle Cardiovascular Imaging Inc., Calgary, Alberta, Canada).

### 1D and 2D echocardiography

Echocardiography was performed using a MX400 ultra-high frequency linear array transducer (18–38 MHz, center transmit: 30 MHz, axial resolution: 50 μm) together with a Vevo® 3100 high-resolution Imaging System (both FUJIFILM VisualSonics, Toronto, Ontario, Canada). Mice were sedated with 3% isoflurane (Baxter International, Deerfield, Illinois, USA) and fixed in dorsal position on a heated pad at 37 °C (FUJIFILM VisualSonics, Toronto, Ontario, Canada), for body temperature maintenance of mice. After depilation, pre-warmed ultrasound gel (Parker Laboratories Fairfield, New Jersey, USA) was applied on the chest. Isoflurane concentration was reduced to a minimum (1–2%) to achieve constant and comparable heart rates during examination (Additional file 1: Table S1).

For 1DE, M-Mode images of the maximum dimension of the LV in parasternal long axis view were acquired as recently described by us [21]. Care was taken to visualize the LV in its maximum dimension from apex to base while recording B-mode images in parasternal long axis view for 2DE analyses. Additionally, velocity profiles of the heart failure cohort of the ascending and descending aorta were carried out using pulsed-wave Doppler mode. All acquired images were digitally stored in raw format (DICOM) for further offline-analyses.

Image analyses were performed by a single observer using the dedicated software package VevoLAB Version 3.0 (FUJIFILM VisualSonics, Toronto, Ontario, Canada). For inter-observer analysis data was analyzed by a second independent observer. Both observers had comparable long-time experience in performing and analyzing small animal echocardiography, including 1DE, 2DE and speckle-tracking echocardiography, but no experience with 3DE.

Cardiac parameters of the heart failure cohort like diastolic wall thicknesses, LV inner diameter (LVID), and fractional shortening (FS) were evaluated in acquired 1DE M-Mode images. LV mass (LVM) was calculated according to the manufacturer’s instructions. Gradient P assessing the degree of aortic stenosis was calculated from velocity parameters 10 weeks post-TAC as described previously [22, 23]. Corresponding 1DE-assessed cardiac volumes and EF were calculated according to the Teichholz formula for both cohorts as followed [24]:

$$ \mathrm{EDV}=\left(\frac{7.0}{2.4+ LVID;d}\right)x\  LVID;{d}^3 $$

$$ \mathrm{ESV}=\left(\frac{7.0}{2.4+ LVID;s}\right)x\  LVID;s3 $$

2DE analysis of both cohorts was determined by using the *LVtrace*-tool of VevoLAB for planimetry in B-Mode images derived from parasternal long axis view. Endocardial borders were traced during end-diastole and end-systole from LV outflow tract to apex. Calculations of 2DE-assessed cardiac volumes and EF were based on monoplane Simpson’s method of discs. All analyses were performed according to the guidelines for cardiac chamber quantification provided by the American Society of Echocardiography [2].

### 3D echocardiography

A detailed standard operating procedure for 3DE can be found in the Online Supplement (Additional file 1). For generation of 3DE datasets, the ultrasound probe was clamped into a specialized 3D-motor (FUJIFILM VisualSonics, Toronto, Ontario, Canada), allowing automated and stepwise movement of the probe. The linear movement of the transducer facilitates image acquisition at multiple levels of the heart with step sizes on a micrometer scale. The parasternal long axis view in maximum dimension from apex to base served as starting point for consecutive image recordings. The system generates 4D data in terms of automatically respiration-gated cine loops to avoid respiratory motion artifacts. Images were recorded with the following settings: scan distance: 0.8–1.2 cm (depending on heart size covering the whole LV); step size: 100 μm, acquisition type: quick; process quality: sharp; frame rate: 200 fps. This resulted in 79–119 scan steps/heart slices and an acquisition time of 3–6 min per animal. All acquired images were digitally stored in raw format (DICOM) for further offline-analyses.

3D-volumes and EF were investigated by multi-slice reconstruction starting the analysis with a picture at maximum expansion of the LV. The distance between analyzed images amounts to 1 mm (Fig. 1D). Manual tracing of the images was performed, leading to 5–8 analyzed images (depending on heart size) at one time point of the cardiac cycle (spatial dimension, 3D). In total, three different time periods of the cardiac cycle (end-diastolic, mid-systolic and end-systolic) (temporal dimension, 4D), automatically chosen by VevoLAB software tool, were analyzed (Fig. 1D). LV volumes and corresponding EF were calculated, using a disc summation without assumptions. Exemplary tracings and 3D reconstructions of the cardiac volume can be found in the Additional files 2, 3 and 4.

For calculation of inter- and intra-observer variabilities, identical echocardiographic images (SHAM: *n* = 7, TAC: *n* = 9) were analyzed with 1DE, 2DE and 3DE by the same observer twice or by another investigator, respectively.

### Statistical analysis

All analyses were performed using GraphPad Prism 7. A *p*-value of < 0.05 was assumed as statistically significant. Results are shown as mean ± standard error of mean (SEM). Normal distribution of variables was verified in advance of further statistical analysis, using the Kolmogorov Smirnov Normality Test. Statistical analyses were performed using unpaired two-tailed Student’s t-test, one-way-ANOVA for multiple comparisons followed by Uncorrected Fisher’s LSD posttest or two-way-ANOVA for multiple comparisons followed by Tukey’s multiple comparisons test, as appropriate. Method comparisons and inter- and intra-observer variabilities were analyzed using Bland-Altman plots. Results of Bland-Altman analysis were expressed as bias and agreement intervals. The rate of agreement was defined by the percent difference to gold standard MRI values (method comparison) or the first observer (inter-observer variability) as follows: ≤ ±5% excellent, ≤ ±10% good, ≤ ±20% moderate, ≤ ±30% poor.

## Results

### Validation of 3DE in vitro

In a first step, we evaluated the accuracy of 3DE in vitro, by assessing the volumes of round-shaped phantoms in comparison to conventional 2DE and MRI as gold standard measurement (Fig. 2). To this end, latex balloons (Fig. 2a) were scanned and analyzed with conventional 2DE (Fig. 2b), novel 3DE tomographic multi-slice reconstructions (Fig. 2c) and MRI (Fig. 2d). 1DE was not applied since the underlying Teichholz formula is based on an ellipsoid geometric shape not being fulfilled by the used round-shaped phantoms [25]. Bland-Altman analysis of 2DE and 3DE in comparison to gold standard MRI measurements revealed that 3DE tended to underestimate phantom volumes, whereas 2DE misestimated in both directions (Fig. 2e, f). This effect might be due to MRI artifacts caused by the agarose gel matrix. Further, 3DE showed good agreement when compared to gold standard MRI measurements, whereas conventional 2DE showed excellent values for mean differences, but large agreement intervals, misestimating strongly in both directions (Fig. 2e, f).

### Validation of 3DE in vivo

**Fig. 3:**
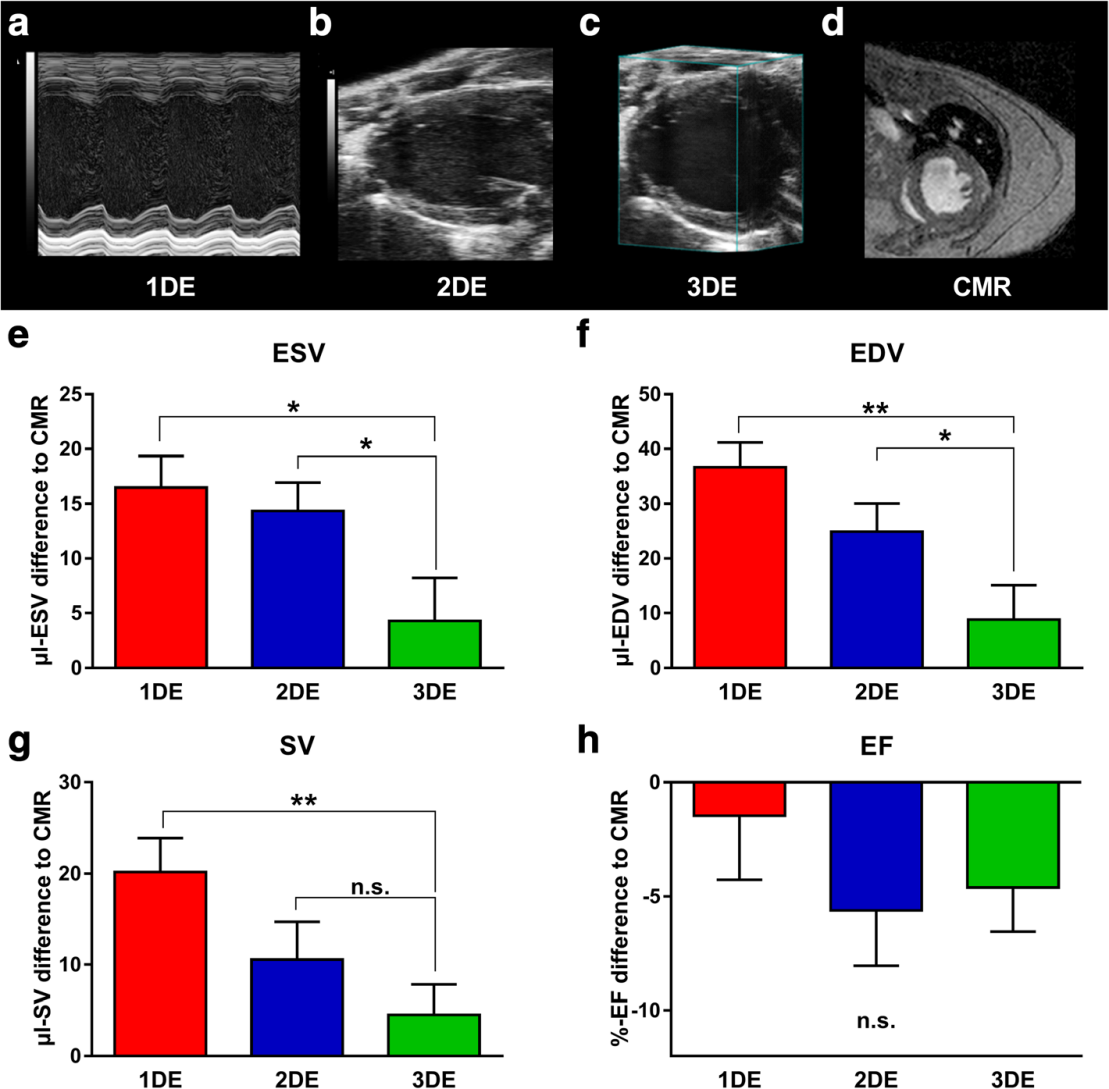
In vivo volumetry**. a** Exemplary 1DE, **b** 2DE, **C** 3DE and **d** cardiac magnetic resonance (CMR) images of left ventricles of NZO mice. Mean differences of echocardiographic-assessed **e** ESV, **f** EDV, **G** SV and **h** EF to values assessed with gold standard CMR (CMR) imaging. *n* = 10. *p < .05, **p < .01 vs. 3DE

In a second step, we validated 3DE against conventional 2DE and cardiac magnetic resonance (CMR) imaging under in vivo conditions in a validation cohort consisting of C57/BL6 and NZO mice, aiming for a broad range of cardiac performance (Table 1). NZO mice are known to develop severe obesity and therefore show increased blood pressure levels, heart and body weights [26, 27]. The cardiac phenotype of NZO mice is reflected by significantly enhanced end-diastolic (EDV) and end-systolic volumes (ESV), stroke volumes (SV) and decreased EFs, when compared to CMR-assessed parameters of healthy control mice (Table 1). In addition to CMR measurements, we applied 1DE, 2DE and novel 3DE to the validation cohort and compared the results to CMR-derived volumetric data (Fig. 3). Figure 3 shows exemplary pictures of the compared imaging modalities A) M-Mode (1DE), B) B-Mode (2DE), C) tomographic multi slice reconstruction (3DE) and D) CMR (Fig. 3a-d). 3DE-assessed ESVs and EDVs showed significant smaller mean differences to CMR-assessed volumes, when compared to 1DE and 2DE-assessed volumes (ESV - 1DE: 16.5 ± 2.9 μl, 2DE: 14.3 ± 2.6 μl, 3DE: 4.3 ± 3.9 μl; EDV – 1DE: 36.7 ± 4.5 μl, 2DE: 24.9 ± 5.2 μl, 3DE: 8.8 ± 6.3 μl) (Fig. 3e, f). Of note, all echocardiographic modalities tended to overestimate the true LV volumes. Based on the values of EDV and ESV the relative measures SV and EF were calculated. SVs assessed by 3DE showed the smallest mean difference to the CMR-assessed SVs (Fig. 3g). Significant differences among the echocardiographic modalities were found between 1DE and 3DE, but not between 2DE and 3DE (Fig. 3g). The clinical relevant measure EF was underestimated by all echocardiographic techniques (Fig. 3h). However, no significant differences were observed between the echocardiographic modalities (Fig. 3h).

**Table 1:** CMR-characteristics of validation cohort

	C57BL/6 J	NZO
n-number (%)	5 (50)	5 (50)
CMR Data		
ESV, μl	13.22 ± 1.9	52.42 ± 16.5*
EDV, μl	34.61 ± 3.1	90.22 ± 18.6*
EF, %	62.5 ± 2.6	47.76 ± 7.5*
SV, μl	21.39 ± 1.4	37.81 ± 2.1***

Mean ± SEM. Student’s t-test. *p < .05; ***p < .001 vs. C57BL/6 J cohort. ESV = end-systolic volume; EDV = end-diastolic volume; EF = ejection fraction; SV = stroke volume

### Application of 3DE in experimental heart failure

**Fig. 4:**
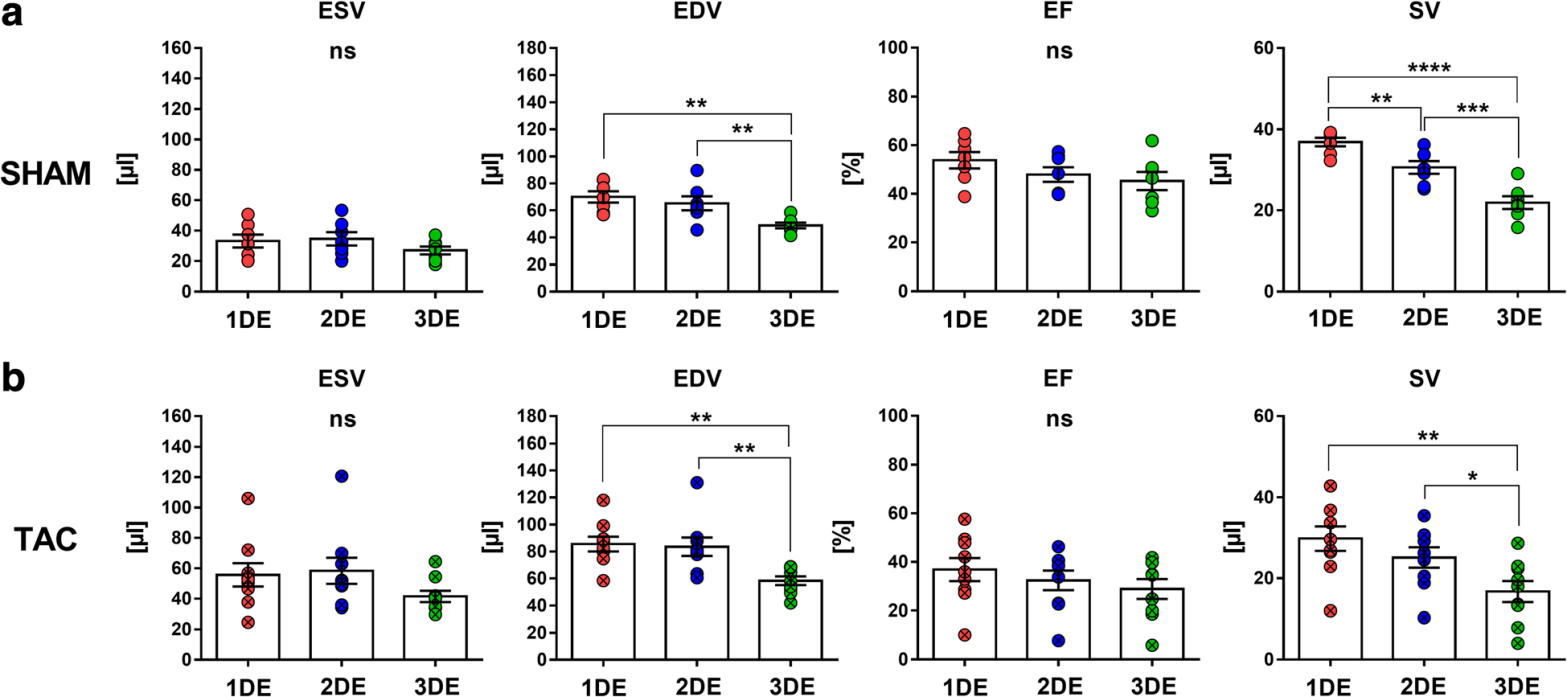
Echocardiographic method comparison in healthy controls and mice with pressure overload-induced heart failure. **a** Statistical comparison of echocardiographic imaging modalities assessing ESV, EDV, SV and EF in SHAM-control mice. **b** Statistical comparison of echocardiographic imaging modalities assessing ESV, EDV, SV and EF in TAC-mice. Mean + SEM. SHAM: *n* = 7, TAC: *n* = 9. *p < .05, **p < .01, ***p < .001, ****p < .0001 vs. 3DE

To test whether novel commercially available 3DE is suitable to detect expected alterations of cardiac performance, we applied 3DE in a standard mouse model of pressure overload-induced heart failure realized by TAC-surgery. Successful TAC-surgery was proven by increased pressure gradients (*Gradient P*) measured across the aortic banding (Table 2). TAC induced a marked cardiac hypertrophy in terms of LV wall thickening and increased internal diameters pointing towards a dilatation of the LV (1DE, M-mode) (Table 2). All echocardiography modalities reliably detected the presence of a significantly reduced EF among TAC-operated animals (Table 3). Additionally, the extent of EF reduction was similar among the methods used (Table 3). Interestingly, however, only 3DE detected a significant increase in EDV after TAC, whereas 1DE and 2DE failed to reach statistical significance (Table 3). All methods detected a significant increase of ESV after TAC without significant differences among the techniques (Table 3). Within SHAM- and TAC-groups, we observed no significant differences of ESV or EF values determined by the different echocardiographic techniques (Fig. 4). In direct comparison of values derived from the different echocardiographic methods, 3DE showed significantly lower EDVs and SVs in healthy mice and TAC mice (independent from disease status) when compared to 1DE and 2DE (Fig. 4).

**Table 2:** Phenotypic characterization of heart failure cohort

	SHAM (n = 7)	TAC (n = 9)
Stenosis		
Aortic peak velocity desc, mm/s	− 945.7 ± 74.4	− 3107 ± 142.4****
Aortic peak velocity asc, mm/s	1200 ± 72.0	1268 ± 139
Gradient P	−2.17 ± 1.1	32.22 ± 3.2****
1DE		
Heart rate, bpm	489.2 ± 24.7	512.5 ± 16.5
LVAW, d, mm	0.61 ± 0.04	0.84 ± 0.03***
LVPW, d, mm	0.63 ± 0.02	0.73 ± 0.02**
LVID, d, mm	3.99 ± 0.1	4.34 ± 0.12*
LVM, mg	66.91 ± 3.2	105.2 ± 5.9***
FS, %	27.68 ± 2.1	17.98 ± 2.5*

Mean ± SEM. Student’s t-test. *p < .05. **p <.01. ***p <.001. ****p <.0001. SHAM: *n* = 7, TAC: *n* = 9. Asc = ascendens; desc = descendens; LVAW, d = left ventricular anterior wall (diastole); LVPW, d = left ventricular posterior wall (diastole); LVID, d = left ventricular inner diameter (diastole); FS = fractional shortening

**Table 3:** Method comparison of imaging modalities in the heart failure cohort

Method	1DE		2DE		3DE	
	SHAM	TAC	SHAM	TAC	SHAM	TAC
ESV, μl	33.15 ± 4.3	55.72 ± 7.6*	34.63 ± 4.4	58.4 ± 8.6*	26.95 ± 2.6	41.57 ± 3.7**
EDV, μl	70.01 ± 4.2	85.53 ± 5.5	65.24 ± 5.2	83.54 ± 6.8	48.84 ± 2.1	58.31 ± 3.2*
EF, %	53.85 ± 3.4	36.89 ± 4.8*	47.96 ± 3.0	32.4 ± 4.1*	45.29 ± 3.8	28.9 ± 4.1*
SV, μl	36.87 ± 1.0	29.8 ± 3.5	30.62 ± 1.6	25.2 ± 2.5	21.89 ± 1.6	16.7 ± 2.6

Mean ± SEM. Student’s t-test. *p <.05. **p <.01. SHAM: n = 7, TAC: n = 9. EDV = end-diastolic volume; ESV = end-systolic volume; EF = ejection fraction; SV = stroke volume

### Reproducibility of measures

**Fig. 5:**
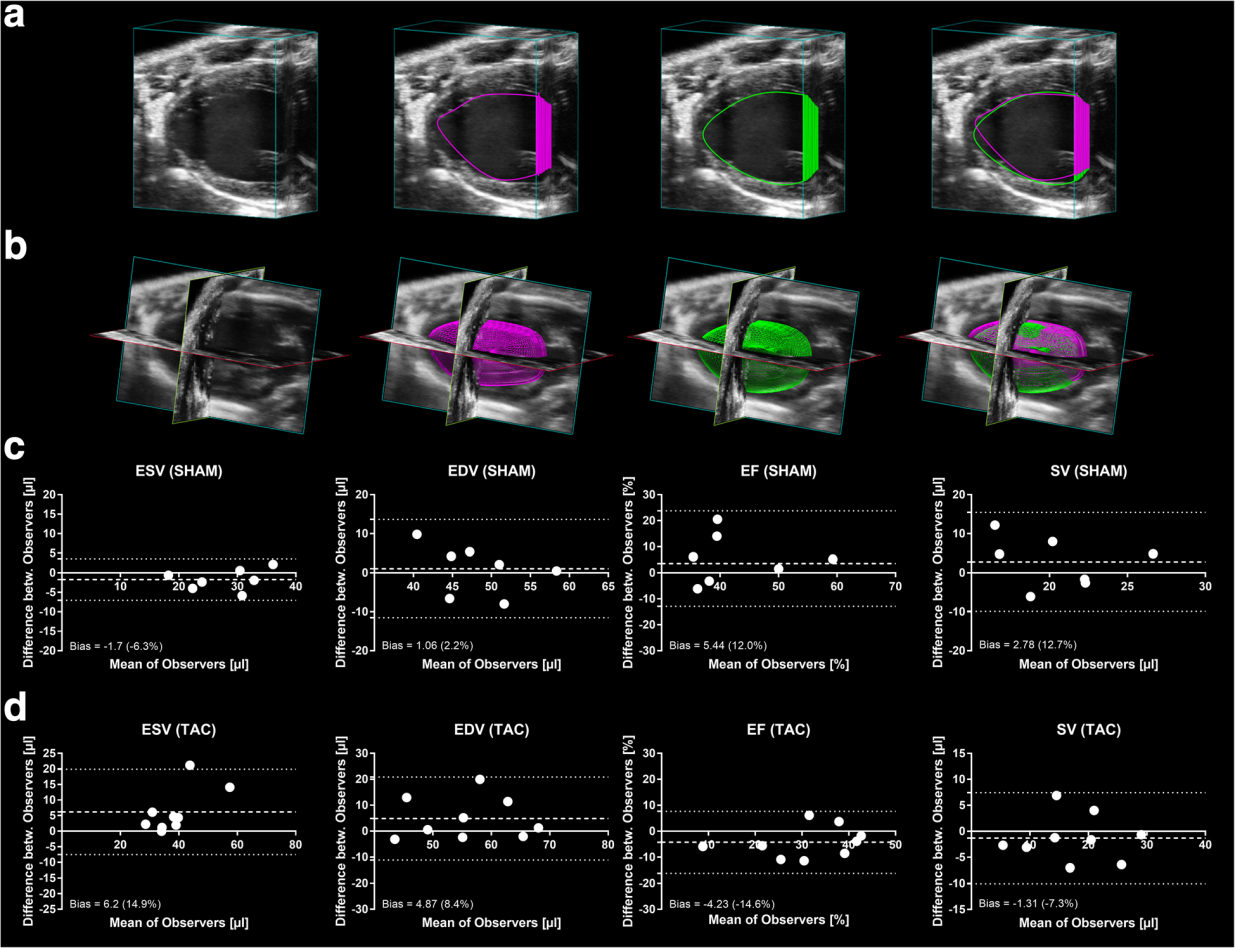
Inter-observer variability of 3DE shown as correlations and corresponding Bland-Altman plots. **a** Representative cube view images of single observer tracings and combined overlay. **b** Representative pictures of all three spatial axes with single observer tracings and combined overlay. **c** Bland-Altman analysis of SHAM (n = 7) and **d** TAC-mice (n = 9) for ESV, EDV, EF and SV showing the inter-observer variability, expressed as absolute difference between observers

All echocardiographic modalities were tested for inter- and intra-observer variability (Table 4). In general, healthy SHAM-mice showed good to excellent inter- and intra-observer variabilities regarding the absolute measures ESV and EDV, whereas TAC-mice demonstrated moderate to good agreement, independent of the echocardiographic technique (Table 4). Further, we observed poorer agreement rates for the relative metrics EF and SV than for total volumes, independent of SHAM or TAC intervention (Table 4). When we compared novel 3D with the conventional echocardiographic techniques 1DE and 2DE, agreement rates for inter-observer variabilities were comparable between imaging modalities, whereas 3DE intra-observer variability appeared to be slightly inferior. Representative planimetric tracings (Fig. 5a) and corresponding reconstructed 3D-volumes (Fig. 5b) of two different observers exemplify the challenge of unambiguous identification of endocardial borders in 3DE. In detail, corresponding Bland-Altman analysis of SHAM and TAC-mice demonstrated good and excellent agreement between observers when analyzing ESV and EDV of healthy SHAM-mice, respectively (Fig. 5c). However, the agreement between observers for the relative metrics of SV and EF was only moderate in healthy mice. When analyzing data of heart failure mice, the inter-observer variability for 3DE metrics was moderate, indicating a difficulty to analyze heart failure mice (Fig. 5d).

**Table 4:** Inter- and intra-observer variabilities

			Inter-observer variability		Intra-observer variability	
			Mean diff. ± SD	LOA	Mean diff. ± SD	LOA
1DE	ESV	SHAM TAC	3.8 ± 6.0 2.8 ± 11.2	−9.4 to 17.0 −20.9 to 26.6	2.3 ± 6.0 − 0.8 ± 11.0	− 10.8 to 15.5 − 24.0 to 22.5
	EDV	SHAM TAC	6.2 ± 6.2 7.2 ± 8.6	− 7.3 to 19.7 − 11.0 to 25.5	2.1 ± 5.8 − 0.3 ± 7.9	− 10.6 to 14.8 − 17.0 to 16.5
	EF	SHAM TAC	−1.4 ± 4.5 2.2 ± 6.5	− 11.2 to 8.4 − 11.5 to 15.9	−2.1 ± 4.3 1.3 ± 5.8	− 11.4 to 7.2 − 11.0 to 13.5
	SV	SHAM TAC	−1.1 ± 5.9 4.4 ± 3.9	−1.1 to 5.9 − 3.8 to 12.6	−0.03 ± 1.5 1.8 ± 4.4	− 3.2 to 3.2 − 7.5 to 11.2
2DE	ESV	SHAM TAC	−1.9 ± 5.7 − 5.4 ± 11.7	− 14.3 to 10.4 − 30.1 to 19.4	0.3 ± 6.2 − 0.5 ± 12.4	− 13.2 to 13.9 − 26.9 to 25.8
	EDV	SHAM TAC	−4.6 ± 6.2 − 7.0 ± 9.2	−18.1 to 9.0 − 26.4 to 12.4	−2.2 ± 7.3 − 0.9 ± 10.0	− 18.2 to 13.7 − 22.0 to 20.3
	EF	SHAM TAC	−1.1 ± 4.6 −0.6 ± 5.9	− 11.1 to 9.0 − 11.9 to 13.1	−2.1 ± 4.3 1.3 ± 5.8	−11.4 to 7.2 − 11.0 to 13.5
	SV	SHAM TAC	−3.9 ± 2.6 − 1.8 ± 3.8	− 9.5 to 1.7 − 9.9 to 6.3	−2.6 ± 2.3 − 0.3 ± 3.7	−7.6 to 2.5 − 8.1 to 7.5
3DE	ESV	SHAM TAC	−1.72 ± 3.5 6.18 ± 4.3	−9.4 to 5.9 − 3.0 to 15.4	−2.6 ± 2.9 4.0 ± 3.9	− 9.0 to 3.7 − 4.3 to 12.2
	EDV	SHAM TAC	1.06 ± 3.6 4.87 ± 4.5	− 6.8 to 8.9 − 4.7 to 14.4	− 0.3 ± 3.4 1.0 ± 4.4	−7.7 to 7.2 − 8.2 to 10.3
	EF	SHAM TAC	5.44 ± 5.3 − 4.23 ± 5.4	−6.2 to 17.1 − 15.7 to 7.2	5.7 ± 4.3 − 5.6 ± 5.4	− 3.7 to 15.1 − 17.1 to 5.9
	SV	SHAM TAC	2.78 ± 2.6 −1.31 ± 3.7	−2.8 to 8.4 − 9.2 to 6.5	2.4 ± 2.2 − 2.9 ± 3.7	− 2.4 to 7.1 − 10.8 to 5.0

SHAM: n = 7, TAC: n = 9. SD = standard deviation; LOA = limits of agreement; ESV = end-systolic volume (μl); EDV = end-diastolic volume (μl); EF = ejection fraction (%); SV = stroke volume (μl)

## Discussion

In the present study, we evaluated a recently launched commercially available 3DE system for small animals in various experimental settings. We were able to show that (1) 3DE-derived volumetry under in vitro conditions is in good agreement with MRI as gold standard measurement; (2) cardiac volume determination with 3DE demonstrates smaller mean differences to CMR-assessed volumes, when compared with conventional echocardiographic techniques; (3) 3DE was suitable to detect reduced EFs in a standard mouse model of pressure overload; (4) Inter- and intra-observer variability of 3DE showed good to excellent agreement regarding absolute volumes in healthy mice, whereas agreement rates for the relative metrics EF and SV demonstrated good to moderate observer variabilities.

Our results in the cardiac phantoms demonstrated that under controlled conditions with clearly delineated boundaries and comparable step sizes between 3DE and MRI, 3DE consistently underestimated phantom volumes, whereas 2DE misestimated in both directions. One explanation for this result might be that tiny air bubbles, emerging at the outer phantom rim within the agarose gel matrix, may cause small MRI artifacts, which consequently generates a halo-like effect during MRI border identification. This would consequently lead to an overestimation of MRI-assessed phantom volumes. A direct method comparison between MRI and echocardiographic-assessed volumes would therefore result in allegedly volume underestimation of echocardiography. However, we only observed consistently underestimation of phantom volumes with 3DE, but not conventional 2DE. We believe that the missing echocardiographic underestimation of 2DE (in comparison to MRI), might be due to the angle-dependency of 2DE, masking the underestimation effect of echocardiography, depending on the positioning of phantoms within the agarose gel matrix and the angle of the transducer. Albeit we cannot prove this hypothetical limitation of 2DE, one of the major advantages of 3DE is to outdistance the angle-dependency of conventional echocardiography and therefore misestimating only in one direction.

Our results demonstrate that 3DE is suitable to determine cardiac volumes in vivo. These findings are in line with pioneering studies, evaluating non-commercially available 3DE-techniques [14, 15]. In 1999, Scherrer-Crosbie and colleagues demonstrated for the first time that multidimensional imaging allows precise LV volumetry and ventricular function, comparable to flow-probe measurements in a mouse model of myocardial infarction [14]. *Dawson* et al. applied ECG- and respiration-gated 3DE in small animals and were the first, who demonstrated excellent agreement by comparison with the current gold standard volumetric technology (MRI) [15]. However, the widespread application of these non-commercially 3DE approaches was limited with regards to standardization, post-processing software and spatial resolutions. Based on these pioneering studies, the present commercially available 3DE system for small animals was launched [16]. Very recently, Damen and colleagues analyzed the novel commercially available 3DE-system in a genetic model of LV hypertrophy and healthy controls in comparison to 1DE and CMR [16]. The authors found no significant differences between 3DE and CMR measured mean values of cardiac volumetry and corresponding relative metrics, whereas 1DE on average overestimated cardiac volumes [16]. In contrast, our results demonstrated a moderate overestimation of 3DE-assessed cardiac volumes when compared to CMR values. This effect might be explained by the differences in step size used for 3DE (step size: 0.1 mm) and CMR (step size: 1.0 mm) analysis in our study. A reduction of CMR-slice thickness will increase spatiotemporal resolution of the acquired images, but will consequently lead to prolonged acquisition time, which further can cause problems with anesthesia. It is known from the clinics that a coarsely chosen resolution of CMR-image lines can lead to partial volume effects, in case the last part of the apex (short axis orientation) is located between two slices and therefore not included during endocardial border tracing [28, 29]. This effect has already been reported for other imaging techniques like positron emission tomography (PET) in preclinical animal models [30, 31]. In terms of our findings, the 10-fold difference in resolution between 3DE and CMR may lead to an ostensible overestimation of 3DE-assessed volumes, but might also be reasoned by a CMR-based partial volume effect. Our findings are in contrast to the data of Damen and colleagues, who detected no significant differences for mean values of cardiac volumes [16], although they also used different slice thicknesses during image acquisition (3DE: 0.076 mm vs CMR: 1.00 mm).

When comparing gold standard CMR to echocardiographic imaging modalities, it turned out that the variability of measurements was lower for absolute cardiac volumes, than for relative metrics. One of the difficulties facing LV functional assessment is that EF varies with changes in blood pressure, heart rate and body temperature [32]. Since major differences regarding physiological and technical conditions between preclinical CMR and echocardiography still exist (e.g. positioning of mice (prone vs. supine position), spatiotemporal resolution (1.0 mm vs. 0.1 mm step sizes)), it seems unlikely to assess identical values for LV volumes with these methods. While our study was designed to keep these parameters constant between different imaging methods by the use of similar anesthesia strategies, especially the difference in positioning of mice between preclinical CMR and echocardiography most probably has a significant impact on hemodynamics that cannot be avoided. Thus, it is mandatory to examine all animals under the same conditions within one modality to increase reproducibility and minimize variations between measurements [32]. However, feasibility of this set up is often restrained by financial and time by financial and temporal requirements. Today, CMR data are widely accepted as the gold standard method for the assessment of cardiac volumes in humans and small animals. Both 3DE and CMR do not rely on geometrical assumptions for formula-based computation of 3D volumes and should therefore be preferred over 1D and 2D methods to avoid inaccuracy when assessing cardiac volumes and function.

A precursor of the present 3DE system has already been validated against Micro-CT and 1DE in a murine model of muscular dystrophy [33]. The authors found that although each aforementioned imaging modality measured decreased cardiac function as disease progresses in genetically modified mice, 3DE had higher agreement with gold standard measurements acquired by gated micro-CT and smaller variability [33]. These data are in line with our findings from the heart failure cohort, showing that all echocardiographic modalities are suitable to detect a decrease of ventricular function, but smallest standard deviation was recognized for 3DE-derived volumetry. In contrast to 1DE and 2DE, 3DE was able to detect expected alterations of EDVs in mice suffering from pressure overload-induced heart failure after TAC-surgery [29]. Cardiac remodeling plays a crucial role during development of heart failure and therefore influences LV volumes [30]. Further, LV volumes were demonstrated as superior predictors of cardiac outcome in heart failure patients, when compared to LVEF [31]. The incremental value of 3DE for the diagnosis of patients has been shown decades ago and became apparent in high accuracy and good feasibility [32]. Especially the diagnoses of cardiac valve diseases and ventricular asynchrony on the basis of LV volume quantification has been demonstrated as great advantage of novel 3DE over conventional echocardiographic approaches in the clinics [7, 33, 34]. Indeed, 3DE used in the clinics is technically based on matrix array transducers, which are currently not available for small animals, hampering the direct translation of results from “bedside to bench”. However, robust assessment of impaired ventricular function, based on altered cardiac volumetry, demonstrates the useful potential of 3DE and the certain advantage over conventional echocardiographic approaches in small animal models.

Further, Bondoc et al. detected only minor standard deviation for 3DE measurements and good reproducibility, while 1DE exhibited considerably greater variability [33]. We found good to moderate inter- and intra-observer variabilities for 3DE, which were comparable or slightly inferior when compared to conventional echocardiography using a different imaging system. This finding might be explained by relevant limitations recognized during image acquisition and analysis of 3DE: The automated image processing algorithm implemented by the VevoLab software does not allow for manual corrections of the chosen time periods for the cardiac cycle or the visualization of endocardial borders for 3DE image acquisition and analysis. In contrast, the analysis of 1DE and 2DE images is based on manual selection of cardiac cycle time periods and also of clearly delineated endocardial borders. In general, automated image processing algorithms are preferred in order to strengthen reproducibility of obtained data sets. However, it appears as a major limitation that the operator cannot verify if the software has chosen the time points for cardiac volume assessment correctly, which also hampers the comparability between conventional echocardiographic imaging modalities and novel 3DE. In addition, the identification of myocardial boundaries in the consecutive tracing seems to be a general and major limitation of the novel 3DE approach. Starting from the maximum dimension of the LV long axis, the problem aggravates when reaching outer regions in which no myocardial borders are visible in most cases. Nevertheless, tracing at these outer slices is required for reconstruction of realistic LV volumes. We included in our study only images with acceptable image quality, enabling us to perform reliable 3DE analysis. A large meta-analysis of 3DE in clinical trials revealed that the inclusion of all 3D datasets, regardless of image quality, increased the variability of 3DE-derived data (as defined by elevated 95% confidence intervals) when compared to studies with pre-selected high image quality [7]. Future studies exclusively focusing on 3DE data sets with high image quality will reveal the impact of image quality on 3DE data in small animal models.

Besides, it should be stressed that valid, precise and robust assessment of cardiac volumetry using a novel software package requires experienced observers, which remains challenging due to the novelty of the imaging technique in small animals. Additionally, a highly standardized protocol for the tracing procedure is required in order to assure comparability between different observers.

In summary, our data indicates that 3DE may provide additional value for basic research, especially in preclinical models in which precise LV volumetry is of interest. However, an extensive evaluation of this currently available commercial 3DE approach is still lacking and only little is known about the ideal field of application. For instance, asymmetric ventricular shape (e.g. after myocardial infarction) represents a major limitation of calculation-based 1DE and 2DE and might be a field of application for 3DE in future [2, 17]. Therefore, 3DE is expected to have add-on value especially when being applied to experimental models in which a non-symmetric LV geometry is expected. Further investigations are required in order to identify suitable indications for usage of 3DE in basic research.

### Limitations

First, all echocardiographic examinations were performed under inhaled anesthesia which might have had an impact on heart rate and function and hampers comparison to CMR-assessed values. Further, echocardiographic examination, including novel 3DE, is always limited due to sternum, rib and lung artifacts, which can blur endocardial borders. Second, the choice of end-diastolic, mid-systolic and end-systolic time periods during the cardiac cycle is automatically done by the VevoLab software. Therefore, the user is dependent on the correct selection with no option for the user to validate the choice of cardiac cycle time periods. This may become relevant when investigating cardiac pathologies with arrhythmias. Third, the sample size of the present study was relatively low and only two animal cohorts were used to evaluate novel 3DE. Thus, future validation using larger sample sizes and different animal models is still required. Fourth, tracing of MRI/CMR data was performed manually, whereas ultrasound images were analyzed with semiautomatic software tools. Fifth, we found moderate inter- and intra-observer variabilities for 3DE in diseased mice, which were comparable or slightly inferior when compared to conventional echocardiography. Sixth, we only acquired images from mice during a single ultrasound session. Future studies will reveal reproducibility of novel 3DE when screening the same animal in multiple ultrasound sessions. Lastly, it should be taken into account that we used a suboptimal setting of body temperature controlling during image acquisition and did not monitor body temperature of mice directly. Therefore, we cannot prove whether body temperature variations had potential confounding effects on the assessment of cardiac volumetry in our study.

## Conclusion

In conclusion, we report here the evaluation of a newly available technique for 3DE in experimental conditions. 3DE-derived volumetry under in vitro conditions was in good agreement with MRI measurements, consistently underestimating phantom volumes. In vivo, 3DE showed smaller mean differences in LV volumes compared to CMR than conventional echocardiography. Further, 3DE was found to be suitable for the detection of altered LV volumes and assessment of impaired cardiac function. The application of 3DE was characterized by rapid acquisition time (compared to CMR), low costs and high spatiotemporal resolutions. However, difficulties with endocardial border tracing and a moderate reproducibility appear as relevant limitations. To achieve the full potential of 3DE for the assessment of LV volumes, further standardization processes for image acquisition and analysis are needed to obtain a valid and robust method, providing a reliable tool for diagnosis of systolic dysfunction.

## Additional files

Additional file 1: Online Supplement. (DOCX 633 kb)

Additional file 2: 4D cine loop of 3-axes without tracing. (AVI 126703 kb)

Additional file 3: 4D cine loop of 3-axes with tracing. (AVI 126703 kb)

Additional file 4: 4D cube view cine loop with tracing. (AVI 24086 kb)

## Abbreviations

1DE: One-dimensional echocardiography
2DE: Two-dimensional echocardiography
3DECMR: Three-dimensional echocardiography cardiac magnetic resonance
EDV: End-diastolic volume
EF: Ejection fraction
ESV: End-systolic volume
FS: Fractional shortening
LOA: Limits of agreement
LV: Left ventricular
LVID: Left ventricular inner diameter
LVM: Left ventricular mass
MRI: Magnetic resonance imaging
NZO: New Zealand Obese
SEM: Standard error of mean
SV: Stroke volume
TAC: Transverse aortic constriction
