# Supplementary material for: Evaluation of a commercial multi-dimensional echocardiography technique for ventricular volumetry in small animals
Source: Cardiovasc Ultrasound. 2018 Jul 3;16:10. doi: 10.1186/s12947-018-0128-9 (PMC6029342; doi:10.1186/s12947-018-0128-9)
Supplement: Supplementary file 1 — Online Supplement. (DOCX 633 kb) [file 12947_2018_128_MOESM1_ESM.docx]

**Table S1** Physiology during image acquisition of both cohorts.

|  | | **Respiratory Rate** | **Heart Rate** |
| --- | --- | --- | --- |
| **Validation cohort** | |  |  |
| **1DE** | C57/Bl6 | 73.2 ± 13.7 | 491.9 ± 18.3 |
|  | NZO | 104.2 ± 7.9** | 512.3 ± 15.6 |
| **2DE** | C57/Bl6 | 62.8 ± 15.1 | 493.2 ± 17.3 |
|  | NZO | 109.2 ± 15.3**^#^ | 516.3 ± 15.0 |
| **3DE** | C57/Bl6 | 65.2 ± 14.3 | 492.2 ± 16.6 |
|  | NZO | 81.6 ± 8.9 | 503.8 ± 15.2 |
| **CMR** | C57/Bl6 | 37.4 ± 3.9 | 473.6 ± 23.1 |
|  | NZO | 42.4 ± 5.9 | 453.2 ± 16.0 |
| **Heart failure cohort** | |  |  |
| **1DE** | SHAM | 43.3 ± 8.3 | 489.2 ± 24.7 |
|  | TAC | 30.3 ± 2.2 | 512.5 ± 16.5 |
| **2DE** | SHAM | 44.4 ± 5.2 | 529.3 ± 13.1 |
|  | TAC | 39.1 ± 3.9 | 515.4 ± 17.1 |
| **3DE** | SHAM | 47.1 ± 13.9 | 472.0 ± 21.8 |
|  | TAC | 31.8 ± 1.3 | 496.6 ± 14.7 |

Mean±SEM. **p < .01 vs. corresponding group during CMR examination. ^#^p < .05 vs. C57/Bl6 group imaged by 2DE. C57/Bl6: n=5, NZO: n=5, SHAM: n=7, TAC: n=9.

**Standard Operating Procedure for 3D-Echocardiography**

Required Material

Imaging System (Vevo©3100, Vevo©2100)

Imaging Station (Vevo Imaging Station)

Transducer (dependent on investigated species)

3D-Motor

Consumables

- Oxygen supply (Linde)
- Isoflurane (Abbot U.S.)
- Isoflurane vaporizer, connected to plastic gas chamber and inhalation mask
- Electrode gel
- Tape
- Depilation crème
- Q-Tips
- Ultrasound gel
- Heater for ultrasound gel
- Infrared lamp

**Installation of 3D-Motor**

Prior to image acquisition, the 3D-Motor has to be installed to the imaging system:

1. Insert the plug of the 3D-Motor into the socket on the back of the imaging system.
2. Install the 3D-Motor to the Vevo imaging station according to the manufacturer’s instructions.
3. Clamp the transducer into the 3D-Motor. Make sure that there is nothing obstructing the 3D-Motor when moving.
4. Initialize the 3D-motor by selecting the button “Motor Initialization” in the Vevo-menu.

**Image Acquisition of a 4D Parasternal Long Axis View of the LV**

1. Prepare a mouse by anesthetizing and depilating it the same way as for 1DE and 2DE image acquisition.
2. Create a B-Mode picture of the parasternal long axis of the LV by rotating the platform 30 degrees to the left. Make sure that the transducer runs precisely parallel to the LV to prevent apex foreshortening.
3. Choose a B-Mode picture of the LV showing the largest dimension of the LV with good visualization of endocardial borders.
4. Minimize the field of vision to minimize amount of data storage.
5. Choose “Motor position” from the Vevo menu.
6. Select “4D Scan” and the settings as desired. We recommend:

| **Settings** | **Recommended** | **Explanation** |
| --- | --- | --- |
| *Scan distance* | < 1 cm | depending on heart size to minimize data volume |
| *Step size* | 100 μm | smaller step size generates better image quality |
| *Acquisition type* | quick | ensures shorter acquisition time |
| *Process quality* | sharp | guarantees good image quality |
| *Frame rate* | 200 fps | lower frame rate reduces quality, higher frame rate improves image quality, but enhances amount of data |

1. Select the green button showing now the number of scan steps and the estimated time of image acquisition (in the recommended example: 79-99 scan steps/heart; acquisition time of 3-4 min per animal):


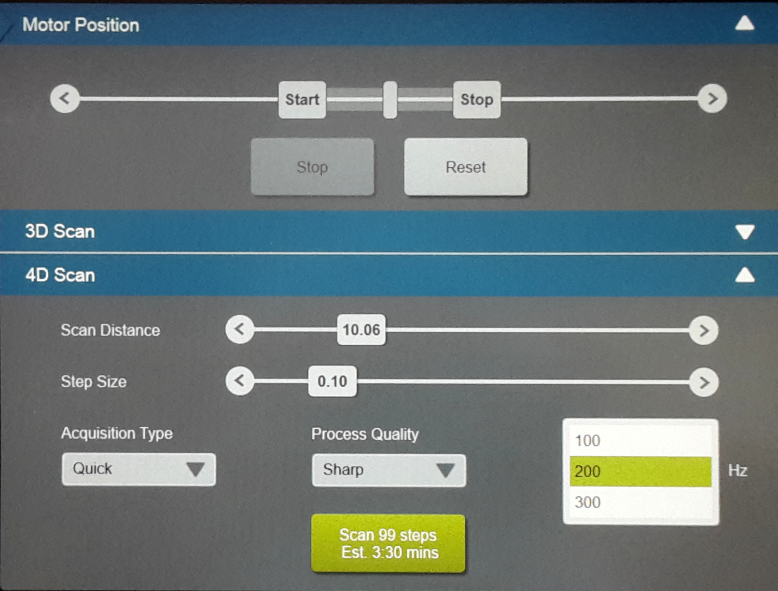


1. 3D-Motor will move automatically through all scan steps.
2. Monitor the anesthesia and heart rate of the mouse precisely to ensure stable image quality during the acquisition process.
3. The reconstruction of the 4D cube view is automatically performed by averaging several B-Mode images acquired via ECG-gating during multiple cardiac cycles.

**Image Analysis**

1. Import acquired study into VevoLAB software.
2. Select image recorded in 4D-mode and choose “Image Processing > Load Into 4D”.
3. A 4D data set is created, which can be displayed in different views (cube view, 3 spatial plane view, single slice view).
4. After clicking “Volume Measurement” different methods can be selected (“Parallel & Rotational Methods” or “Multi-slice Method”).
5. Choose “Multi-slice Method” and click “Start”. The software gives instructions for all following required steps.
6. “Draw the first contour on the volume at the first time point”
7. Select one of the displayed single-slice images for the first tracing.
   1. Start with an image derived from the maximum LV dimension.
   2. Ensure high contrast for clearly visible endocardial borders.
8. Begin your tracing by placing 2 points at the LV outflow track; place the 3rd point at the apex with maximum distance from LV outflow tract; place 2-4 further points on the endocardial border until the ventricular shape is captured properly.
9. This procedure has to be repeated in further single-slice images at the same time point of the cardiac cycle.
10. At least the most outer single-slice images have to be captured.
11. Tracings of images lying in between analyzed images are extrapolated automatically by the software.
12. We recommend manual tracing in at least 5 single-slice images distributed equally over the whole LV.
13. Press “Done” to complete tracing at the first time point of the cardiac cycle.
14. Step 21-24 have to be repeated at middle and quarter time point of the cardiac cycle.
15. Press “Finish” to complete tracings.
16. Functional parameters (EDV, ESV, EF, CO, SV) can be displayed by clicking “Report”.
